# The self-nanoemulsifying drug delivery system of *Petiveria alliacea* extract reduced the homeostatic model assessment-insulin resistance value, interleukin-6, and tumor necrosis factor-α level in diabetic rat models

**DOI:** 10.14202/vetworld.2021.3229-3234

**Published:** 2021-12-31

**Authors:** Arifa Mustika, Nurmawati Fatimah, Gadis Meinar Sari

**Affiliations:** 1 Department of Anatomy, Histology, and Pharmacology, Faculty of Medicine, Universitas Airlangga, East Java, Indonesia; 2 Department of Physiology of Faculty Medicine, Universitas Airlangga, East Java, Indonesia

**Keywords:** diabetes, homeostatic model assessment-insulin resistance, tumor necrosis factor-α, interleukin-6, nanoemulsifying, *Petiveria alliacea*

## Abstract

**Background and Aim:**

Metaflammation plays a significant role in the pathogenesis, development, and complication of diabetes mellitus (DM). This inflammation is associated with insulin resistance. Therefore, the inflammatory pathways have been targeted for pharmacological treatment. *Petiveria alliacea* can decrease blood glucose levels and has anti-inflammatory and antioxidant activities; however, there are still insufficient data regarding its efficacy for the treatment of DM. This study aimed to investigate the effect of the self-nanoemulsifying drug delivery system (SNEDDS) of *P. alliacea* leaf extract on the homeostatic model assessment (HOMA)-insulin resistance (IR) value and interleukin (IL)-6 and tumor necrosis factor-α (TNF-α) levels in a streptozotocin (STZ)-induced diabetic rat model.

**Materials and Methods:**

Thirty-five diabetic rat models were randomly divided into five groups. The first group received the SNEDDS of *P. alliacea* leaf extract at a dose of 50 mg/kg body weight (BW), the second group received it at a dose of 100 mg/kg BW, the third group received it at a dose of 200 mg/kg BW, the fourth group received 18 mg of metformin, and the fifth group only received the SNEDDS formula. The treatment was administered once a day, orally, for 14 days. On the 15^th^ day after treatment, the rats were sacrificed to obtain blood samples for cardiac examination. The IL-6, TNF-α, and insulin levels in the serum were measured using the enzyme-linked immunosorbent assay method. The HOMA-IR value was calculated using a formula.

**Results:**

The mean IL-6 and TNF-α levels were low in the group that received the SNEDDS of *P. alliacea* leaf extract. There was no significant difference in the insulin level in all treatment and control groups. However, a significant difference in the HOMA-IR value was noted between the group that received the SNEDDS of *P. alliacea* leaf extract and metformin and the group that did not receive treatment (p<0.05).

**Conclusion:**

The SNEDDS of *P. alliacea* leaf extract reduced the HOMA-IR value and suppressed the TNF-α and IL-6 levels in the STZ-induced diabetic rat model.

## Introduction

Diabetes mellitus (DM) is a multifactorial metabolic disorder affecting the glucose status of the human body [1,2]. There is a crucial link between metabolic disorder and inflammation, leading to metaflammation. Metaflammation is a systemic and chronic low-grade inflammation associated with excess nutrients and energy. Previous studies [3,4] have demonstrated that diabetes, both types 1 DM (T1DM) and 2 (T2DM), is an inflammatory disease. T1DM occurs due to cell-mediated autoimmunity damage to pancreatic β-cells. T2DM has been regarded as a metabolic disease, so that the metabolic determinant is the main pathogenetic factor. However, more recent research has begun to focus on low-grade inflammation as a significant factor in the pathogenesis, development, and complication of T2DM [4,5]. T2DM is characterized by decreased insulin secretion and increased insulin resistance (IR), associated with chronic inflammation [6,7]. Evidence shows that the adipose tissue in diabetic patients secretes various inflammatory cytokines, such as tumor necrosis factor-α (TNF-α) and interleukin (IL)-6. Increased inflammatory cytokines can predispose the liver to activate the IkB kinase (IKK) activity. Activated IKK inhibits the expression of insulin receptor 1 (IRS-1) substrate, resulting in an increase in IR [8]. Long-term exposure to hyperglycemia leads to an imbalance in oxidative stress, endoplasmic reticulum stress, hypoxia, amyloid and lipid deposition, lipotoxicity, and glucotoxicity. This process can stimulate an immune response to secrete various inflammatory mediators and is associated with systemic IR. Macrophages migrate to the adipose tissue, liver, and muscle in diabetics and obese patients. The macrophages produce pro-inflammatory factors, such as nitric oxide, reactive oxygen species, prostaglandin E2, TNF-α, IL-1b, and IL-6, which induce inflammation. They also increase complications such as atherosclerosis, impaired lung function, and cardiovascular disease [9,10]. As evidence suggests that inflammation plays a significant role in its pathogenesis, T2DM is now redefined as an immune disorder [4,11]. Therefore, the inflammatory pathways involved in T2DM have been targeted for pharmacological manipulation to suppress cytokine inflammation and combat IR.

*Petiveria alliacea* is a perennial plant widely found in Indonesia and empirically used as a folk medicine to treat various diseases, such as diabetes, bleeding cough, anti-inflammation, and immunomodulatory. Several studies revealed that *P. alliacea* has anti-inflammatory [12-15] and antioxidant activities [16]. All parts of *P. alliacea* are available for use in treatment from oxidative damage of cells [17]. Mustika *et al* [18]. showed that the extract of *P. alliacea* leaves decreased the blood glucose level in the T2DM mouse model and increased the 5′-adenosine monophosphate-activated protein kinase (AMPK) expression in the liver [16]. These studies provide hope that *P. alliacea* may be used to control the inflammation in hyperglycemic conditions, thereby reducing IR. One of the disadvantages of *P. alliacea* leaf extract is that it contains complex active compounds, which may compete in the absorption process, resulting in a decline in the absorption of active compounds in the gastrointestinal tract [19,20]. Therefore, to overcome this problem, *P. alliacea* extract formulated in the form of a self-emulsifying drug delivery system (SNEDDS) is made [21]. The evaluation of the SNEDDS formulation of *P. alliacea* leaf extract on the diabetes treatment has not yet been clearly elucidated.

Therefore, this study aimed to investigate the effect of the SNEDDS of *P. alliacea* leaf extract on the homeostatic model assessment (HOMA-IR) value and IL-6 and TNF-α levels in the streptozotocin (STZ)-induced diabetic rat model.

## Materials and Methods

### Ethical approval

This study was approved by the Animal Care and Use Committee of the Faculty of Veterinary Medicine, Universitas Airlangga, Indonesia (No. 2.KE-092.05.2018).

### Study period and location

This study was conducted for 5 months (May-September 2019). Male Wistar rats were reared in the Laboratory Animal of Pharmacology Department, Faculty of Medicine, Universitas Airlangga. The SNEDDS of *P. alliacea* leaf extract preparation was carried out at the Pharmacology Laboratory, Faculty of Medicine, Universitas Airlangga. ELISA of insulin, IL-6, and TNF-α were performed at the Institute of Tropical Diseases, Universitas Airlangga, Surabaya.

### Plant material

*P. alliacea* leaves were collected from Balai Materia Medika, Batu, Indonesia. Subsequently, 1000 g of *P. alliacea* leaf powder was extracted with 70% ethanol solvent. The extraction was performed by maceration for 3 days. The SNEDDS formulation was formed from virgin coconut oil, Tween 80, and propylene glycol. The SNEDDS of *P. alliacea* leaf extract was made by adding *P. alliacea* leaf extract to the SNEDDS formulation [21].

### Chemical

About 70% alcohol, 10% sucrose solution, virgin coconut oil, Tween 80, and polyethylene glycol were purchased from Brataco Co., Ltd. (Indonesia). The enzyme-linked immunosorbent assay (ELISA) kits for IL-6 (catalog no.: EK306/2), TNF-α (catalog no.: EK382/2), and insulin were purchased from Multi Sciences. STZ and metformin were purchased from Sigma-Aldrich^®^ (Singapore) and Kimia Farma^®^ (Indonesia), respectively.

### Animal models

Male *Rattus norvegicus* strain Wistar rats aged 3-4 months old were used in the experiment. The rats weighed 150-200 g and were in good health. They were obtained from the Pharmacological Department, Faculty of Medicine, Universitas Airlangga.

The experimental animals were adapted to the environment for 7 days and fed *ad libitum*. The diabetic rat models were created by inducing these rats with STZ [22-25]. The dose of STZ was 50 mg/kg body weight (BW) and administered by intraperitoneal injection. The rats were given 10% sucrose solution during the first night after induction to avoid sudden hypoglycemia post-injection. Three days after induction, the rats were checked for blood glucose levels using a glucometer. The rats were declared to have diabetes if their blood glucose levels were ≥200 mg/dL and were called the diabetic rat model [18,26-28].

### Experimental treatment

Thirty-five diabetic rat models were randomly divided into five groups. The first group received the SNEDDS of *P. alliacea* leaf extract at a dose of 50 mg/kg BW, the second group received it at a dose of 100 mg/kg BW, the third group received it at a dose of 200 mg/kg BW, the fourth group received 18 mg of metformin, and the fifth group only received the SNEDDS formula.

The treatment was administered once a day, orally, for 14 days. Before and after treatment, the rats were weighed. The rats were sacrificed on the 15^th^ day after therapy to obtain blood samples for cardiac examination. The IL-6, TNF-α, and insulin levels in the serum were measured using the ELISA method.

### Cytokines and insulin examination

Serum samples were collected from all groups and diluted according to the manufacturer’s instructions in the kit. The IL-6, TNF-α, and insulin levels were measured by the ELISA kit (Multi Sciences).

### HOMA-IR value

The HOMA-IR value was calculated using the formula from Qu *et al*. [29], that is, fasting insulin level (mIU/mL) multiplied by the fasting blood glucose level (mmol/L)/22.5. The HOMA-IR value is used to determine IR. The HOMA-IR values were categorized as follows: ≤2.60, normal; 2.60-3.80, borderline; and >3.80, a high correlation with IR [29-31].

### Statistical analysis

Statistical analysis was performed using the SPSS Statistics for Windows, Version 17.0. (IBM Corp., NY, USA). The values were analyzed by one-way analysis of variance followed by the least significant difference *post hoc* multiple comparison test. p<0.05 was considered statistically significant.

## Results

### The effect of STZ administration on the blood glucose level

The mean blood glucose levels in rats before and 3 days after STZ administration were 104.5 and 397.2 mg/d, respectively. These data suggest that STZ administration can increase the blood glucose level in rats.

### The effect of the SNEDDS of *P. alliacea* leaf extract on the IL-6 and TNF-α levels in the serum diabetic rat model

**Figure-1:**
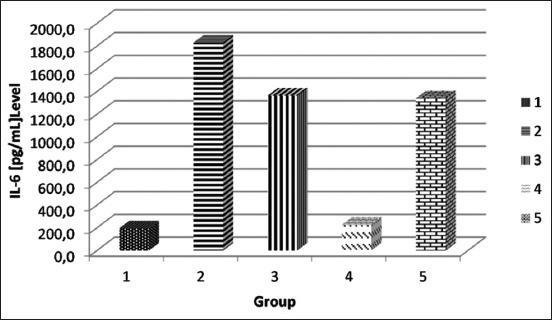
The mean interleukin (IL)-6 level in the serum diabetic rat model. Group 1 received the self-nanoemulsifying drug delivery system (SNEDDS) of *Petiveria alliacea* leaf extract at a dose of 50 mg/kg body weight (BW), Group 2 received it at a dose of 100 mg/kg BW, Group 3 received it at a dose of 200 mg/kg BW, Group 4 received metformin suspension at a dose of 18 mg, and Group 5 received the SNEDDS formula.

The results of the IL-6 levels in the diabetic rat serum are shown in Figure-1. The results of the TNF-α levels in the diabetic rat serum are shown in Table-1. The statistical analysis showed significant differences in the mean TNF-α level between the groups that received either the SNEDDS of *P. alliacea* leaf extract or metformin treatment and the groups that did not receive treatment (p<0.05). The IL-6 level also showed a significant difference between the group that received the SNEDDS of *P. alliacea* leaf extract at a dose of 50 mg/kg BW and metformin and the group that did not receive treatment (p<0.05).

**Table 1:** The mean tumor necrosis factor-α levels in the diabetic rat models.

Group	Mean	Minimum	Maximum	Standard deviation
1	29.4	22.04	38.30	5.58561
2	26.9	8.72	44.96	11.97433
3	24.2	15.78	47.29	11.85768
4	26.1	14.36	44.96	10.16328
5	6268.3	35.66	13,619.88	7250.39135

Group 1 received the self-nanoemulsifying drug delivery system (SNEDDS) of *Petiveria alliacea* leaf extract at a dose of 50 mg/kg BW, Group 2 received it at a dose of 100 mg/kg BW, Group 3 received it at a dose of 200 mg/kg BW, Group 4 received metformin suspension at a dose of 18 mg, and Group 5 received the SNEDDS formula

### The effect of the SNEDDS of *P. alliacea* leaf extract on the serum insulin level and HOMA-IR in the diabetic rat model

**Figure-2:**
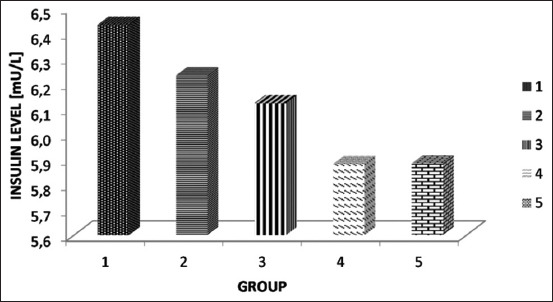
The mean insulin level in the serum diabetic rat model. Group 1 received the self-nanoemulsifying drug delivery system (SNEDDS) of *Petiveria alliacea* leaf extract at a dose of 50 mg/kg body weight (BW), Group 2 received it at a dose of 100 mg/kg BW, Group 3 received it at a dose of 200 mg/kg BW, Group 4 received metformin suspension at a dose of 18 mg, and Group 5 received the SNEDDS formula.

**Figure-3:**
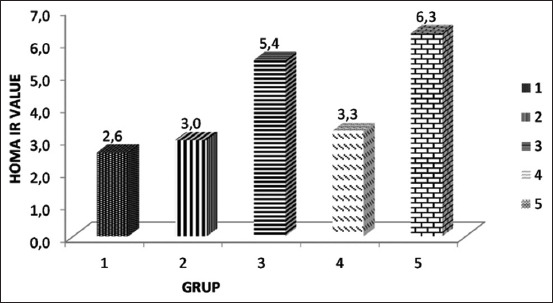
The mean HOMA-IR value in the diabetic rat model. Group 1 received the self-nanoemulsifying drug delivery system (SNEDDS) of *Petiveria alliacea* leaf extract at a dose of 50 mg/kg body weight (BW), Group 2 received it at a dose of 100 mg/kg BW, Group 3 received it at a dose of 200 mg/kg BW, Group 4 received metformin suspension at a dose of 18 mg, and Group 5 received the SNEDDS formula.

The mean insulin level in the serum diabetic rat model is shown in Figure-2, and the mean HOMA-IR value is shown in Figure-3. Based on the HOMA-IR value in the untreated group (6.8), it showed that induction using STZ can cause IR. In the group that received metformin therapy, the HOMA-IR value was 3.3, indicating a borderline level, while in the group that received the SNEDDS of *P. alliacea* leaf extract at doses of 50 and 100 mg/kg BW, the HOMA-IR values were 2.6 and 3, indicating normal and borderline levels, respectively.

There was no significant difference in the insulin level in all treatment and control groups; however, a significant difference in the HOMA-IR value was noted between the groups that received the SNEDDS of *P. alliacea* leaf extract and metformin and the group that did not receive treatment (p<0.05).

## Discussion

This study shows that the induction of STZ at the dose of 50 mg/kg BW by intraperitoneal injection can cause hyperglycemic conditions, IR (a HOMA-IR value of 6.8) and an increase in the pro-inflammatory mediators, namely, TNF-α and IL-6. These data support the concept of metaflammation, which is a chronic low-level inflammatory condition. This process is highly relevant for the development of diabetes and its complications. This condition contributes to the development of T2DM by increasing IR in peripheral tissues, especially in the liver, muscle, and adipose tissue, and causes damage to the pancreas, which will interfere with insulin secretion [32].

Hyperglycemic conditions can lead to inflammation. Cytokines or markers of inflammation that increase during such conditions include C-reactive protein, TNFa, IL-1, and IL-6 [2,10]. The inflammatory factor can decrease insulin signaling by phosphorylating IRS-1 on some serine residues, and consequently, it decreases the ability of IRS-1 to perform insulin signal transduction. IR is a major defect underlying the development of T2DM and has been defined in the last decade as a metaflammation disease [4]. TNF-α interferes with insulin signal transduction and affects carbohydrate metabolism in cells and tissues. The mechanisms of TNF-α to interfere with insulin signal transduction may be the downregulation of the IR expression and IRS-1 substrate [9,10]. The cytokine IL-6 decreases hepatic insulin sensitivity by activating the pro-inflammatory pathway and concurrently inhibiting IRS signaling [33]. This study is also in line with the research by Bayat *et al* [9]. and Sharma *et al* [31]. exhibiting that induction with STZ in *R. norvegicus* showed the same characteristics of an increased cytokine level and IR [33]. Haidari *et al*. [33] showed that the increased levels of TNF and IL-6 are closely related to the occurrence of IR. This study shows a relationship between hyperglycemia, inflammation, and IR. Hyperglycemia can cause inflammation, inflammatory conditions will cause IR, and IR conditions will increase blood glucose levels. This circle will worsen the condition of DM and cause complications.

This study demonstrated that the SNEDDS of *P. alliacea* leaf extracts at a dose between 50 and 200 mg/kg BW can significantly reduce the IL-6 and TNF-α levels in the diabetic rat models. This study also showed that the insulin levels in the group of rats that received SNEDDS of *P. alliacea* leaf extracts are higher than those in the control group. This result is in accordance with the HOMA-IR value, where the group that received the SNEDDS of *P. alliacea* leaf extracts has a normal HOMA-IR value. The data showed that the SNEDDS of *P. alliacea* leaf extracts can increase insulin sensitivity in a diabetic rat model.

Based on the results of this study, the mechanism of action of the SNEDDS of *P. alliacea* leaf extract in increasing insulin sensitivity is presumably because the extract can reduce the IL-6 and TNF-α levels. TNF-α inhibits insulin signaling by increasing serine/threonine phosphorylation of IRS-1. IL-6 inhibits insulin signaling by decreasing tyrosine phosphorylation [8]. In addition, the decreased level of inflammatory cytokines causes a decrease in the activation of c-Jun N-terminal kinases (JNKs) so that there is no inhibition of IRS-1. JNKs are members of the mitogen-activated protein kinase superfamily and one of the most studied signal transducers in IR. The JNK signaling pathway can be activated by various factors, one of which is cytokines. JNK activation negatively affects insulin signaling pathways leading to IR. A decreased JNK activity also reduces the activation of the nuclear factor kappa-light-chain-enhancer of activated B cells (NF-κB) signaling pathway, causing the decrease in the transcription of inflammatory cytokines [34-36].

The mechanisms of the SNEDDS of *P. alliacea* leaf extracts to reduce the IL-6 and TNF-α levels require further study. However, our previous research on *P. alliacea* leaf extracts proved that the extract could increase the AMPK-a1 expression in the liver of the diabetic rat models [18]. Therefore, the possible mechanism of action of the SNEDDS of *P. alliacea* leaf extracts is the reduction in the IL-6 and TNF-α levels through AMPK activation. AMPK is a promising target for DM treatment. When AMPK is activated, it will affect various metabolic processes, such as glucose metabolism, lipid metabolism, and mitochondrial metabolism, and it plays a significant role in inflammation, autophagy, and apoptosis [32,37].

The activation of the AMPK pathway decreases the function of the NF-κB system, whereas the NF-κB signaling pathway is a major pathway involved in the activation of both the innate and adaptive immune systems [32,35,38]. The process of inhibiting the NF-κB signaling pathway will inhibit the transcription of inflammatory genes so that the synthesis of IL-6 and TNF-α cytokines decreases [25,32,34]. Moreover, the AMPK activation inhibits the monocyte chemoattractant protein 1 (MCP-1) secretion [39].

The NF-κB signaling pathway also regulates IL-8, chemokines, and adhesion molecules, such as MCP-1, intercellular adhesion molecule 1 (ICAM-1), and vascular cell adhesion protein 1 (VCAM-1) [40,41]. Several studies have shown that an increase in the serum MCP-1 level is correlated with IR. MCP-1 also initiates the migration and proliferation of ICAM-1 and VCAM-1 [39,40]. These mediators play a pivotal role in the development of the vascular complication of diabetes [41]. Exploration of other pro-inflammatory cytokines and chemokines will be intriguing, but this step could not be performed in this study due to our limitations. Therefore, further research is needed to determine the role and mechanism of *P. alliacea* as therapy in DM.

A drug with a pleiotropic effect is the current approach to diabetes treatment. *P. alliacea* is a medicinal plant with various properties, such as lowering blood glucose levels, increasing AMPK expression, and reducing IR and pro-inflammatory cytokine levels. These data indicate that *P. alliacea* has potential as a therapy in DM because it has a pleiotropic effect.

## Conclusion

Our study showed that the SNEDDS of *P. alliacea* leaf extract reduced the HOMA-IR value and suppressed the TNF-α and IL-6 levels in the STZ-induced diabetic rat model.

## Authors’ Contributions

AM: Conceptualized the study, conducted the experiment and laboratory examination, analyzed the data, and wrote and revised the manuscript. NF: Prepared the SNEDDS of *P. alliacea* leaf extract, analyzed the data, and wrote the manuscript. GMS: Analyzed the data and edited the manuscript. All authors read and approved the final manuscript.

## References

[1] Oguntibeju O.O (2019). Medicinal plants and their effects on diabetic wound healing. Vet. World.

[2] Tsalamandris S, Antonopoulos A.S, Oikonomou E, Papamikroulis G.A, Vogiatzi G, Papaioannou S, Deftereos S, Tousoulis D (2019). The role of inflammation in diabetes:Current concepts and future perspectives. Eur. Cardiol.

[3] Zhong J, Gong Q, Mima A (2017). Inflammatory regulation in diabetes and metabolic dysfunction. J. Diabetes Res.

[4] De Candia P, Prattichizzo F, Garavelli S, De Rosa V, Galgani M, Di Rella F, Spagnuolo M.I, Colamatteo A, Fusco C, Micillo T, Bruzzaniti S, Ceriello A, Puca A.A, Matarese G (2019). Type 2 diabetes:How much of an autoimmune disease?. Front. Endocrinol.

[5] Tilg H, Moschen A.R (2008). Inflammatory mechanisms in the regulation of insulin resistance. Mol. Med.

[6] Liu C, Lv L, Guo W, Mo L, Huang Y, Li G, Huang X (2018). Self-nanoemulsifying drug delivery system of tetrandrine for improved bioavailability:Physicochemical characterization and pharmacokinetic study. BioMed. Res. Intern.

[7] Guo R, Liu B, Wang K, Zhou S, Li W, Xu Y (2014). Resveratrol ameliorates diabetic vascular inflammation and macrophage infiltration in db/db mice by inhibiting the NF-κB pathway. Diab. Vasc. Dis. Res.

[8] Liu Y.N, Jung J.H, Park H, Kim H.S (2014). Olive leaf extract suppresses messenger RNA expression of proinflammatory cytokines and enhances insulin receptor substrate 1 expression in the rats with streptozotocin and high-fat diet-induced diabetes. Nutr. Res.

[9] Bayat E, Dastgheib S, Egdar S, Mokarram P (2017). Effect of the aquatic extract of stevia on the serum level of interleukin-6 in streptozotocin-nicotinamide induced diabetic rats. Shiraz E-Med J.

[10] Shen S.C, Chang W.C, Chang C.L (2012). Fraction from wax apple (*Syzygium samarangense* (Blume) Merrill and Perry) fruit extract ameliorates insulin resistance via modulating insulin signaling and inflammation pathway in tumor necrosis factor a-treated FL83B mouse hepatocytes. Int. J. Mol. Sci.

[11] Tsai S, Clemente-Casares X, Revelo X.S, Winer S, Winer D.A (2015). Are obesity-related insulin resistance and Type 2 diabetes autoimmune diseases?. Diabetes.

[12] Oluwa A, Avoseh O, Omikorede O, Ogunwande I, Lawal O (2017). Study on the chemical constituents and anti-inflammatory activity of essential oil of *Petiveria alliacea* L. Br. J. Pharmacol. Res.

[13] Sathiyabalan G, Evanjaline M, Muthukumarasamy S, Mohan V.R (2018). Anti-inflammatory activity of whole plant of *Petiveria alliacea* L. (Phytolaccaceae). Int. J. Pharm. Sci. Rev. Res.

[14] Gutierrez R.M.P, Hoyo-Vadillo C, Hoyo-Vadillo C (2017). Anti-inflammatory potential of *Petiveria alliacea* on activated RAW264.7 murine macrophages. Pharmacogn. Mag.

[15] Alves T.C, Rodrigues E, Lago J.H.G, Prado C.M, Girardi C.E.N, Hipólide D.C (2019). *Petiveria alliacea*, a plant used in Afro-Brazilian smoke rituals, triggers pulmonary inflammation in rats. Rev. Bras. Farmacogn.

[16] Gunawan V.A, Soetjipto H, Mustika A (2020). Hypoglycemic and antioxidant activity of *Petiveria alliacea* in diabetic rat models. Health Sci. J.

[17] Olomieja A.O, Olanrewaju I.O, Ayo-Ajayi J.I, Jolayemi G.E, Daniel U.O, Mordi R.C (2021). Antimicrobial and antioxidant properties of *Petiveria alliacea*. IOP Conf. Earth Environ. Sci.

[18] Mustika A, Indrawati R, Sari G.M (2017). Effect of *Petiveria alliacea* leaves extract in decreasing serum level of blood glucose level through activation of AMPK-a1. in Indonesian J. Clin. Pharm.

[19] Kumar R, Sharma M (2018). Herbal nanomedicine interactions to enhance pharmacokinetics, pharmacodynamics, and therapeutic index for better bioavailability and biocompatibility of herbal formulations. J. Nanosci.

[20] Watkins R, Wu L, Zhang C, Davis R.M, Xu B (2015). Natural product-based nanomedicine:Recent advances and issues. Int. J. Nanomed.

[21] Mustika A, Fatimah N, Sari G.M (2019). Formulation and characterizations of self-nanoemulsifying drug delivery system of extract *Petiveria alliacea* (Singawalang) leaves. Int. J. Appl. Pharm.

[22] Purwanto B, Wiyasihati S.I, Masyitha P.A, Wigati K.W, Irwadi I (2019). Golden sea cucumber extract revives glucose transporter-4 and interleukin-6 protein level in diabetic mouse muscle. Vet. World.

[23] Kavitha K, Reddy A.G, Reddy K.K, Kumar C.S, Boobalan G, Jayakanth K (2016). Hypoglycemic, hypolipidemic and antioxidant effects of pioglitazone, insulin and synbiotic in diabetic rats. Vet. World.

[24] Qosimah D, Aryani D.E, Beltran M.A.G, Aulanni'am A (2019). Diabetes sepsis on Wistar rat strain (*Rattus norvegicus*) induced by streptozotocin and bacteria *Staphylococcus aureus*. Vet. World.

[25] Suryadiningrat M, Kurniawati D.Y, Mujiburrahman A, Purnama M.T.E (2021). Dietary polyvinyl alcohol and alginate nanofibers ameliorate hyperglycemia by reducing insulin and glucose-metabolizing enzyme levels in rats with streptozotocin-induced diabetes. Vet. World.

[26] Lukačínová A, Hubková B, Rácz O, Ništiar F (2013). Animal Models for Study of Diabetes Mellitus, Diabetes Mellitus Insights and Perspectives, Oluwafemi O. Oguntibeju. IntechOpen, London.

[27] Dewinta N.R, Mukono I.S, Mustika A (2020). Pengaruh pemberian ekstrak dandang gendis (*Clinacanthus nutans*) terhadap kadar glukosa darah pada tikus wistar model diabetes melitus. J. Med. Vet.

[28] Handoko A, Purwanto B, Mustika A (2017). The effect of eccentric activity on glucose transporter Type 4. in gastrocnemius muscle of streptozotocin-induced diabetes mellitus mice. J. Agromed. Med. Sci.

[29] Qu H.Q, Li Q, Rentfro A.R, Fisher-Hoch S.P, McCormick J.B (2011). The definition of insulin resistance using HOMA-IR for Americans of Mexican descent using machine learning. PLoS One.

[30] Sukarno D.A, Mustika A, Rejeki P.S (2020). Effect of celery extract on fructose induced insulin resistance *Rattus norvegicus*. Folia Med. Indones.

[31] Sharma A.K, Bharti S, Kumar R, Krishnamurthy B, Bhatia J, Kumari S, Arya D.S (2012). *Syzygium cumini* ameliorates insulin resistance and β-cell dysfunction via modulation of PPARg, dyslipidemia, oxidative stress, and TNF-α in Type 2 diabetic rats. J. Pharmacol. Sci.

[32] Peixoto C.A, Oliveira W.H, Araújo S.M.D, Nunes A.K.S (2017). AMPK activation:Role in the signaling pathways of neuroinflammation and neurodegeneration. Exp. Neurol.

[33] Haidari F, Zakerkish M, Karandish M, Saki A, Pooraziz S (2016). Association between serum Vitamin D level and glycemic and inflammatory markers in non-obese patients with Type 2 diabetes. Iran. J. Med. Sci.

[34] Hotamisligil G.S (2017). Foundations of immunometabolism and implications for metabolic health and disease. Immunity.

[35] Feng J, Lu S, Ou B, Liu Q, Dai J, Ji C, Zhou H, Huang H, Ma Y (2020). The role of JNK signaling pathway in obesity-driven insulin resistance. Diabetes Metab. Syndr. Obes.

[36] Solinas G, Becattini B (2017). JNK at the crossroad of obesity, insulin resistance, and cell stress response. Mol. Metab.

[37] Zhao C, Yang C, Wai S.T.C, Zhang Y, Portillo M.P, Paoli P, Wu Y, San Cheang W, Liu B, Carpéné C, Xiao J, Cao H (2019). Regulation of glucose metabolism by bioactive phytochemicals for the management of Type 2 diabetes mellitus. Crit. Rev. Food Sci. Nutr.

[38] Salminen A, Hyttinen J.M.T, Kaarniranta K (2011). AMP-activated protein kinase inhibits NF-κB signaling and inflammation:Impact on healthspan and lifespan. J. Mol. Med. (Berl).

[39] Mancini S.J, Boyd D, Katwan O.J, Strembitska A, Almabrouk T.A, Kennedy S, Palmer T.M, Salt I.P (2018). Canagliflozin inhibits interleukin-1b-stimulated cytokine and chemokine secretion in vascular endothelial cells by AMP-activated protein kinase-dependent and independent mechanisms. Sci. Rep.

[40] Liu T, Zhang L, Joo D, Sun S.C (2017). NF-κB signaling in inflammation. Signal Transduct. Target. Ther.

[41] Lin Y, Ye S, He Y, Li S, Chen Y, Zhai Z (2018). Short-term insulin intensive therapy decreases MCP-1 and NF-κB expression of peripheral blood monocyte and the serum MCP-1 concentration in newly-diagnosed Type 2 diabetics. Arch. Endocrinol. Metab.

